# The flexibility–complementarity dichotomy in receptor–ligand interactions†

**DOI:** 10.1039/c4sc03398a

**Published:** 2014-12-15

**Authors:** Hongmei Sun, Christopher A. Hunter, Eva Marina Llamas

**Affiliations:** a Department of Chemistry, University of Sheffield Sheffield S3 7HF UK herchelsmith.orgchem@ch.cam.ac.uk; b Department of Chemistry, University of Cambridge Lensfield Road Cambridge CB2 1EW UK

## Abstract

Synthetic supramolecular complexes provide an opportunity for quantitative systematic exploration of the relationship between chemical structure and molecular recognition phenomena. A family of closely related zinc porphyrin–pyridine complexes was used to examine the interplay of conformational flexibility and geometric complementarity in determining the selectivity of molecular recognition events. The association constants of 48 zinc porphyrin–pyridine complexes were measured in two different solvents, toluene and 1,1,2,2-tetrachloroethane (TCE). These association constants were used to construct 32 chemical double mutant cycles to dissect the free energy contributions of intramolecular H-bonds between the phenol side arms of the porphyrins and the ester or amide side arms of the pyridine ligands. Effective molarities (EM) for the intramolecular interactions were determined by comparison with the corresponding intermolecular H-bonding interactions. The values of EM do not depend on the solvent and are practically identical for amide and ester H-bond acceptors located at the same site on the ligand framework. However, there are variations of an order of magnitude in EM depending on the flexibility of the linker used to connect the H-bond acceptors to the pyridine ligands. Rigid aromatic linkers give values of EM that are an order of magnitude higher than the values of EM for the corresponding ester linkers, which have one additional torsional degree of freedom. However, the most flexible ether linkers give values of EM that are also higher than the values of EM for the corresponding ester linkers, which have one less torsional degree of freedom. Although the penalty for conformational restriction on binding is higher for the more flexible ether linkers, this flexibility allows optimization of the geometric complementarity of the ligand for the receptor, so there is a trade off between preorganization and fit.

## Abstract 2

Binding affinity does not increase uniformly with preorganization, because there is a trade off between flexibility and fit.

## Graphical abstract

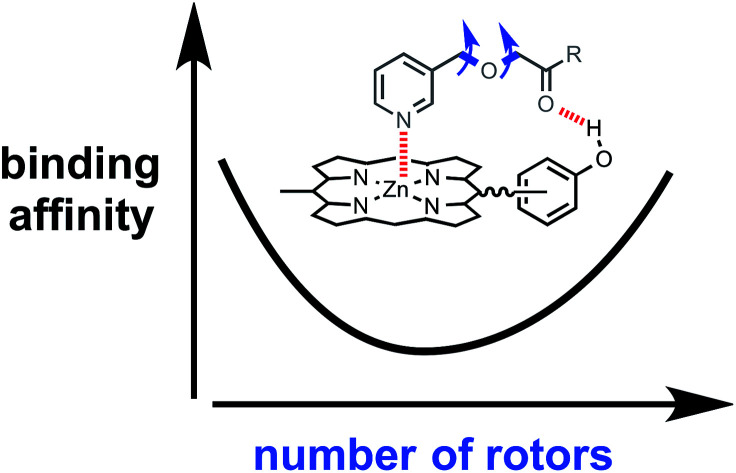

## Introduction

The principle of preorganization proposed by Cram suggests that any complexation-induced reduction in conformational mobility reduces binding affinity.^1^ It is entropically unfavorable to shift numerous conformations available to flexible hosts and guests to the limited conformational ensemble required for optimal binding,^2^ so perfect conformational complementarity between a rigid host and a rigid guest can lead to extremely high binding affinities.^3^ For example, Anderson showed that complexes of a cyclic zinc porphyrin oligomer with multivalent ligands were much more stable than the corresponding complexes of linear porphyrin oligomers.^4^ In these systems, preorganization leads to a remarkable increase of four orders of magnitude in binding affinity. However, the design of perfect complementarity in a rigid host–guest complex is challenging, because such systems are less tolerant of subtle geometric mismatches compared with more flexible systems.^5^ Nature uses flexible molecules that fold up to achieve high affinity binding, and this strategy has been adopted in the area of foldamers to make synthetic hosts.^6^ These results suggest that an alternative way to obtain high binding affinity is to use flexible hosts or guests that undergo cooperative conformational changes upon binding.^7^

We have been developing families of closely related zinc porphyrin–pyridine complexes to quantify the detailed relationship between chemical structure and cooperativity, which governs the behaviour of supramolecular systems.^8^ Fundamental structure–activity studies of this type will provide quantitative design rules to guide the construction of supramolecular receptors and assemblies. In a previous study, we investigated the thermodynamic advantages of freezing out a rotor to preorganize one of the components of a zinc porphyrin–pyridine complex.^9^ In a series of sixteen different supramolecular architectures, we found that the stabilities of the complexes consistently increased by about 5 kJ mol^−1^ when a rotor was removed from the ligands. This result indicates that preorganization offers a significant thermodynamic advantage in terms of receptor–ligand binding affinity and is consistent with results obtained from a range of different experiments in the literature.^3*i*,10^ To test the generality of this principle, we have now designed a new set of ligands with increased conformational flexibility. Extrapolation of the results above would suggest that addition of a new rotor to the ligand framework should *decrease* binding affinity by a further 5 kJ mol^−1^. However, we show here that this is not the case. For a series of sixteen different supramolecular architectures, addition of a rotor consistently *increases* binding affinity by about 3 kJ mol^−1^, which indicates that the relationship between conformational flexibility, preorganization and binding affinity is far from simple.

## Approach

**Fig. 1:**
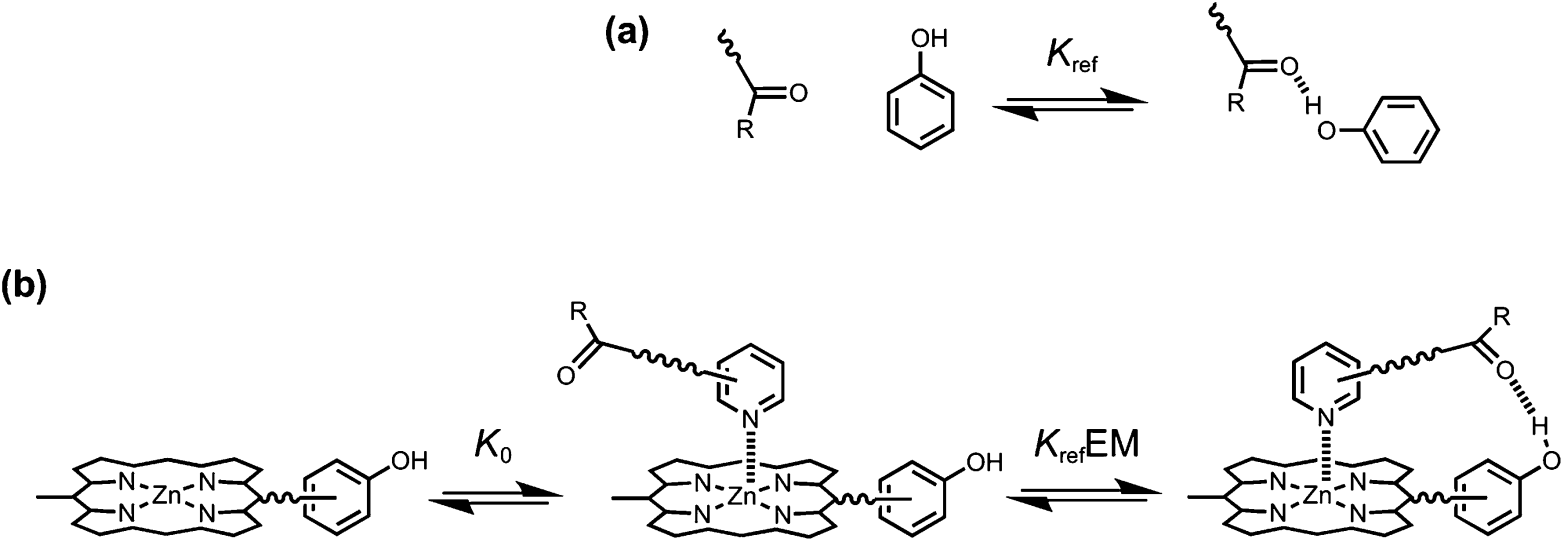
(a) Formation of an intermolecular H-bond. (b) Stepwise equilibria in the formation of a porphyrin–pyridine complex containing an intramolecular H-bond. *K*_ref_ is the association constant for formation of the corresponding intermolecular H-bond. *K*_0_ is the intermolecular association constant for formation of the zinc–nitrogen interaction. *K*_ref_EM is the association constant for formation of the intramolecular H-bond, and EM is the effective molarity for the intramolecular interaction. Some bonds and substituents on the porphyrin are not shown for clarity.

The key parameter that is used to quantify chelate cooperativity is effective molarity (EM), which measures the thermodynamic advantage of an intramolecular interaction compared with the corresponding intermolecular interaction.^11^ Measurement of EM using synthetic porphyrin–pyridine complexes is illustrated in Fig. 1. If we consider stepwise formation of the zinc–nitrogen coordination bond and the H-bond in the complex in Fig. 1b, then *K*_ref_EM represents the association constant for formation of an intramolecular interaction, where *K*_ref_ is the association constant for formation of the corresponding intermolecular interaction (Fig. 1a). Strictly, the H-bond in Fig. 1b is a second intermolecular interaction, but in this paper, we will refer to this interaction as intramolecular, because it governs the second step of the process shown in Fig. 1b. By comparing the intramolecular (*K*_ref_EM) and intermolecular (*K*_ref_) association constants for H-bond formation, the effective molarity (EM) can be experimentally determined.

**Fig. 2:**
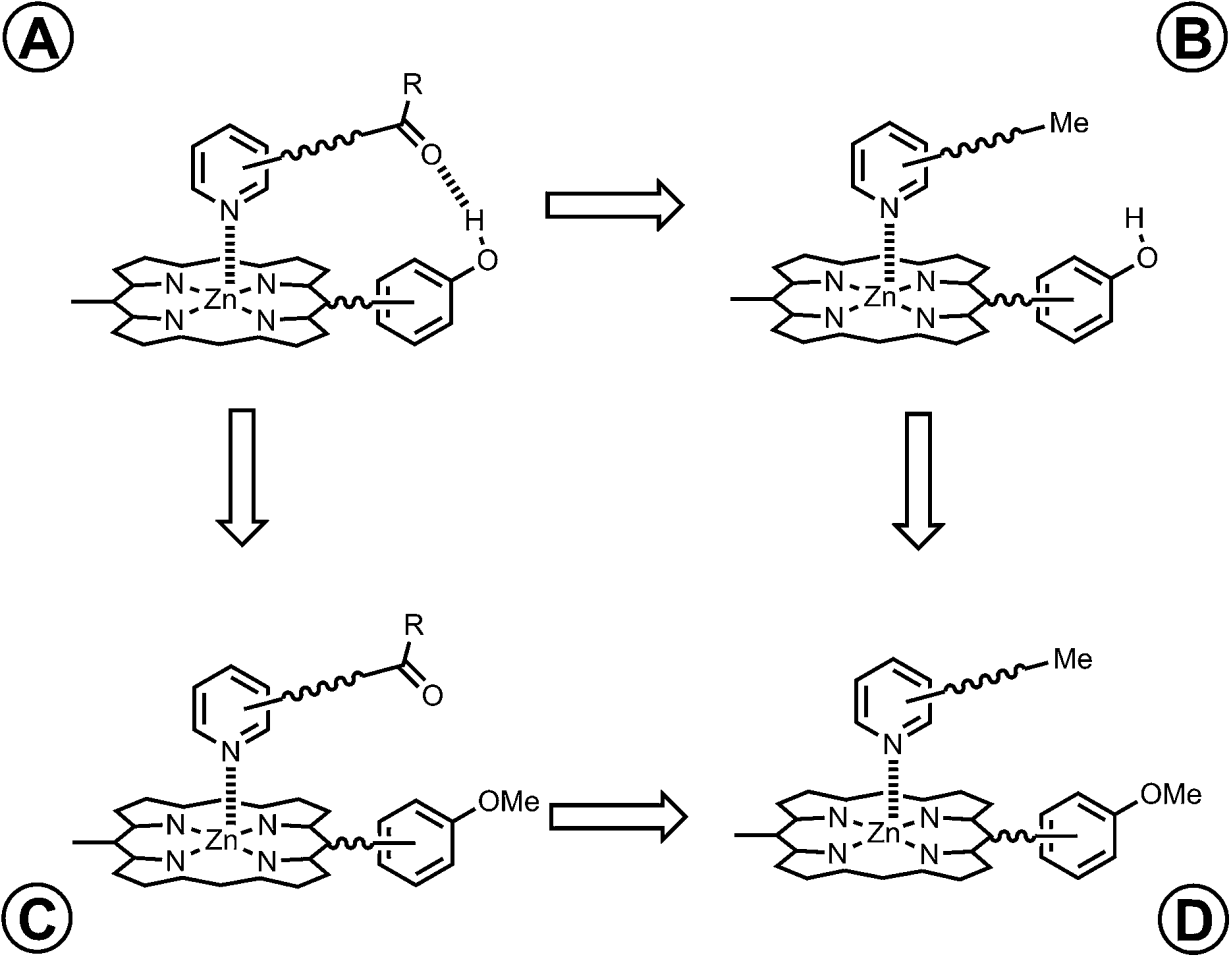
Chemical double mutant cycle (DMC) for measurement of the free energy contribution of an intramolecular H-bond to the stability of complex A.

However, the intramolecular association constant *K*_ref_EM cannot be measured directly. We therefore use a chemical double mutant cycle (DMC) to dissect out the free energy contribution of an intramolecular H-bond from the overall stability of a complex (Fig. 2).^11*d*,12^ Complex A in Fig. 2 is held together by a coordination bond and a H-bond. Complex B has no H-bond, so the difference between the free energy changes for formation of complexes A and B provides a measurement of the free energy contribution of the H-bond in complex A. However, when the H-bond acceptor is removed from the ligand, there are secondary effects. For example, there might be a change in the zinc–nitrogen interaction, so a control experiment is needed to measure this effect. The difference between the free energy changes for formation of complexes C and D measures change in the zinc–nitrogen interaction in a system that does not make a H-bond. Thus the difference between the A to B mutation and the C to D mutation allows us to dissect out the free energy contribution of the intramolecular H-bond to the overall stability of complex A using eqn (1). This approach accounts for all changes in secondary interactions, which cancel in a pairwise manner in the DMC (assuming that free energy contributions are additive).^8*a*,11*d*,12^ The free energy change measured by the DMC, ΔΔ*G*^0^, can then be used to determine the EM for the intramolecular interaction using eqn (2).^8*a*^

1 ΔΔ*G*^0^ = Δ*G*^0^_A_ − Δ*G*^0^_B_ − Δ*G*^0^_C_ + Δ*G*^0^_D_

2 ΔΔ*G*^0^ = −*RT* ln(1 + *K*_ref_EM)

**Fig. 3:**
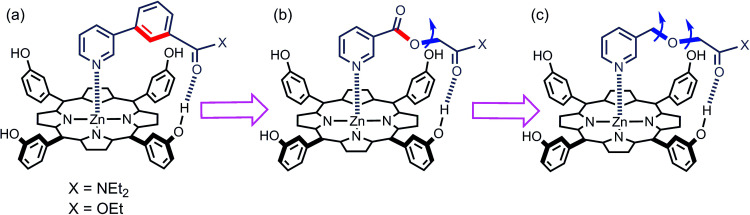
Zinc porphyrin complexes with pyridine ligands that have a H-bond acceptor located at the same position on the ligand framework but varying degrees of conformational flexibility (a) rigid linker, (b) one additional rotor, and (c) two additional rotors. The key rotatable bonds are highlighted in blue, and the restricted rotors are highlighted in red.

We have used this approach to study the effect of conformational restriction on supramolecular effective molarities using families of complexes exemplified in Fig. 3a and b. Compared with the ligand in Fig. 3b, the ligand in Fig. 3a has one less degree of conformational freedom in the linker which connects the H-bond acceptor to the ligand. To explore the role of conformational flexibility further, we have developed new family of more flexible ligands exemplified by the complex illustrated in Fig. 3c. All three ligand families have a H-bond acceptor located at identical positions on the framework but a different number of rotors in the linker. Comparison of EM values for the three ligand families in 48 different supramolecular architectures provides new insight into the relationship between conformational flexibility, geometric complementarity and chelate cooperativity.

## Results and discussion

**Fig. 4:**
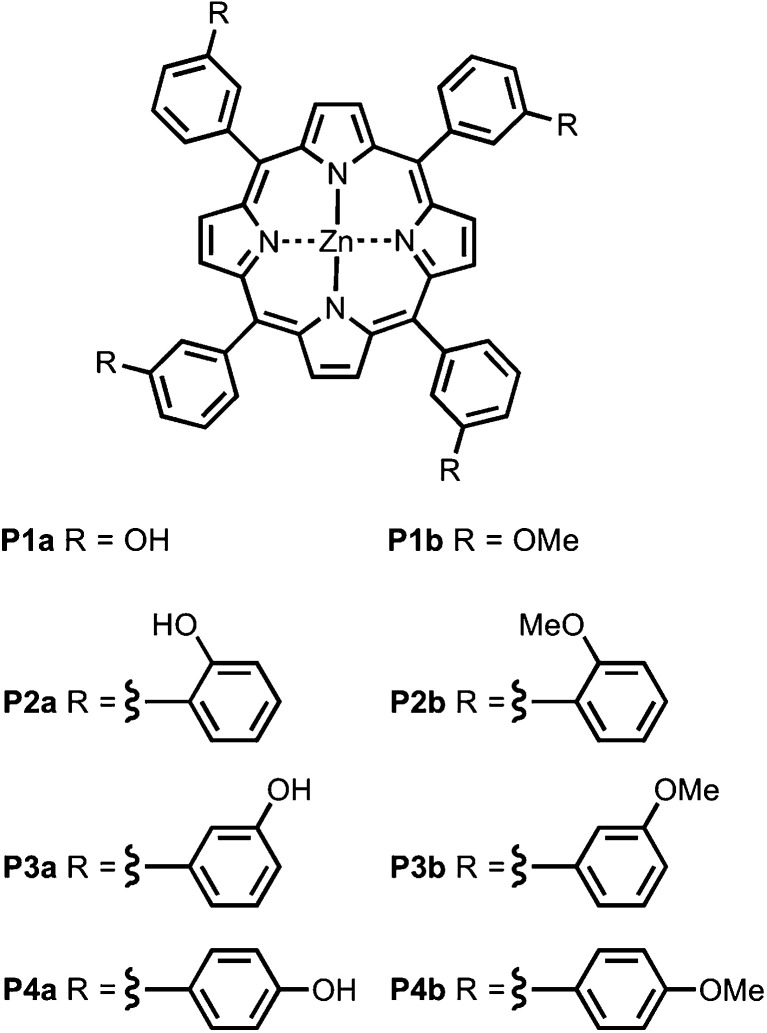
Porphyrin receptors that can make H-bonds (P1a–P4a), and that cannot (P1b–P4b).

**Fig. 5:**
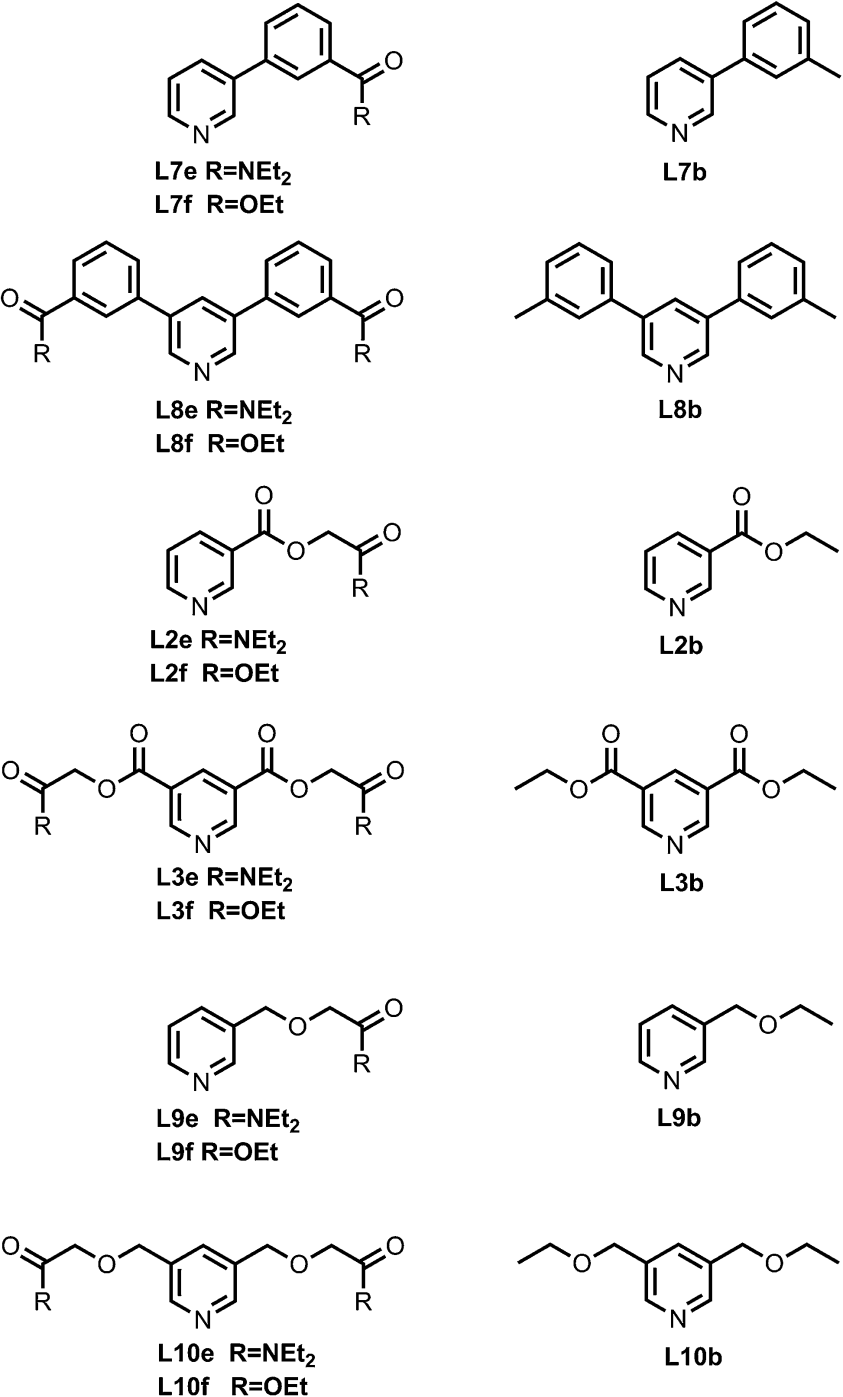
Pyridine ligands equipped with amide (Lf) or ester (Le) H-bonds acceptors and the corresponding control ligands with no H-bonding groups (Lb).

Fig. 4 shows the structures of porphyrin receptors used in this work. Porphyrins P1a–P4a have peripheral phenol H-bond donor groups at different locations, and P1b–P4b are the corresponding non-H-bonding controls with methoxy groups. The three ligand families (aromatic linker, ester linker and ether linker) are shown in Fig. 5. The ligands are equipped with two different H-bond acceptor groups, amide (Le) and ester (Lf). The corresponding control ligands (Lb), which cannot make H-bonds, are also shown.

### Synthesis

**Scheme 1:**
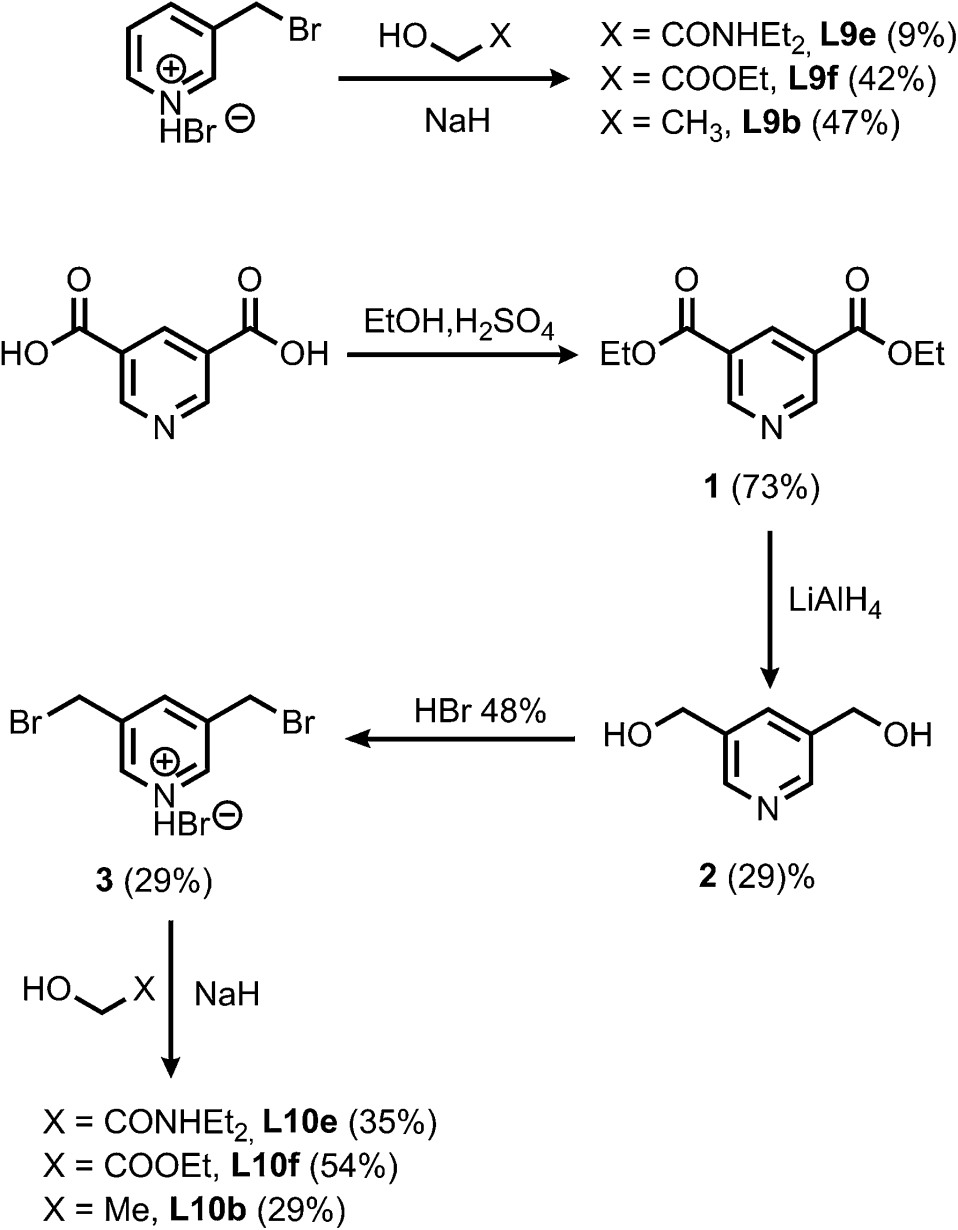


The synthesis of the porphyrin receptors and the ligands with ester (L2 and L3) and aromatic linkers (L7 and L8) was published previously.^9^ The ligands with ether linkers (L9 and L10) were synthesized by coupling the corresponding alcohol with 3-bromomethyl pyridine hydrobromide or 3,5-dibromomethyl pyridine hydrobromide (3), which was prepared from pyridine 3,5-dicarboxylic acid (Scheme 1).^13^

### Binding studies

The association constants for formation of the 48 complexes between the 8 porphyrins and the 6 ether ligands (L9 and L10) were measured using UV/Vis absorption titrations and fluorescence titrations both in toluene and in TCE (see Experimental section for details). All titration data fit well to a 1 : 1 binding isotherm, and the results are listed in Tables 1 and 2 for toluene and TCE respectively. Association constants for the complexes formed by the L2, L3, L7 and L8 ligand families in toluene and in TCE have been reported previously.^9^

**Table 1:** Association constants (*K*/M^−1^) for the formation of 1 : 1 complexes in toluene at 298 K (with percentage errors in brackets)

Ligand	Porphyrin							
	P1a	P2a	P3a	P4a	P1b	P2b	P3b	P4b
L9b	6.8 × 10^3^ (30%)	1.1 × 10^4^ (6%)	1.5 × 10^4^ (3%)	1.2 × 10^4^ (10%)	7.3 × 10^3^ (5%)	5.5 × 10^3^ (7%)	1.1 × 10^4^ (5%)	8.9 × 10^3^ (8%)
L10b	7.1 × 10^3^ (10%)	1.3 × 10^4^ (5%)	1.9 × 10^4^ (5%)	1.1 × 10^4^ (6%)	1.3 × 10^4^ (20%)	6.1 × 10^3^ (6%)	1.3 × 10^4^ (5%)	1.1 × 10^4^ (3%)
L9f	1.5 × 10^4^ (5%)	1.1 × 10^4^ (5%)	2.5 × 10^4^ (5%)	6.1 × 10^3^ (3%)	5.3 × 10^3^ (6%)	3.8 × 10^3^ (5%)	7.5 × 10^3^ (5%)	5.8 × 10^3^ (3%)
L10f	7.7 × 10^4^ (10%)	2.4 × 10^4^ (5%)	9.9 × 10^4^ (8%)	7.8 × 10^3^ (3%)	7.4 × 10^3^ (40%)	4.1 × 10^3^ (4%)	9.9 × 10^3^ (3%)	8.2 × 10^3^ (5%)
L9e	1.8 × 10^5^ (6%)	1.4 × 10^5^ (5%)	4.8 × 10^5^ (7%)	1.1 × 10^4^ (7%)	5.5 × 10^3^ (30%)	3.8 × 10^3^ (3%)	7.4 × 10^3^ (3%)	6.0 × 10^3^ (5%)
L10e	1.7 × 10^7^ (20%)^a^	1.9 × 10^6^ (10%)^a^	2.5 × 10^7^ (10%)^a^	4.1 × 10^4^ (20%)	9.4 × 10^3^ (40%)	5.1 × 10^3^ (4%)	1.2 × 10^4^ (5%)	1.0 × 10^4^ (4%)

a Measured by manual fluorescence titration.

**Table 2:** Association constants (*K*/M^−1^) for the formation of 1 : 1 complexes in TCE at 298 K (with percentage errors in brackets)

Ligand	Porphyrin							
	P1a	P2a	P3a	P4a	P1b	P2b	P3b	P4b
L9b	2.5 × 10^3^ (10%)	4.5 × 10^3^ (5%)	3.3 × 10^3^ (6%)	2.4 × 10^3^ (20%)	2.4 × 10^3^ (30%)	1.8 × 10^3^ (6%)	2.6 × 10^3^ (6%)	2.3 × 10^3^ (4%)
L10b	2.0 × 10^3^ (20%)	3.4 × 10^3^ (3%)	2.9 × 10^3^ (3%)	1.9 × 10^3^ (6%)	1.6 × 10^3^ (20%)	1.3 × 10^3^ (4%)	2.4 × 10^3^ (10%)	1.6 × 10^3^ (3%)
L9f	6.4 × 10^3^ (10%)	2.8 × 10^3^ (5%)	3.4 × 10^3^ (3%)	1.1 × 10^3^ (5%)	8.6 × 10^2^ (10%)	7.6 × 10^2^ (3%)	9.8 × 10^2^ (3%)	9.8 × 10^2^ (5%)
L10f	2.2 × 10^4^ (40%)	2.6 × 10^3^ (8%)	4.0 × 10^3^ (10%)	7.1 × 10^2^ (4%)	7.8 × 10^2^ (20%)	5.7 × 10^2^ (3%)	7.5 × 10^2^ (20%)	6.8 × 10^2^ (4%)
L9e	4.2 × 10^4^ (5%)	1.1 × 10^4^ (3%)	2.0 × 10^4^ (4%)	1.4 × 10^3^ (5%)	9.2 × 10^2^ (9%)	8.5 × 10^2^ (3%)	1.1 × 10^3^ (3%)	1.1 × 10^3^ (7%)
L10e	6.5 × 10^5^ (30%)	5.3 × 10^4^ (20%)	1.1 × 10^5^ (7%)	1.8 × 10^3^ (10%)	4.0 × 10^2^ (4%)	3.5 × 10^2^ (3%)	8.5 × 10^2^ (5%)	4.2 × 10^2^ (10%)

### DMC analysis

**Fig. 6:**
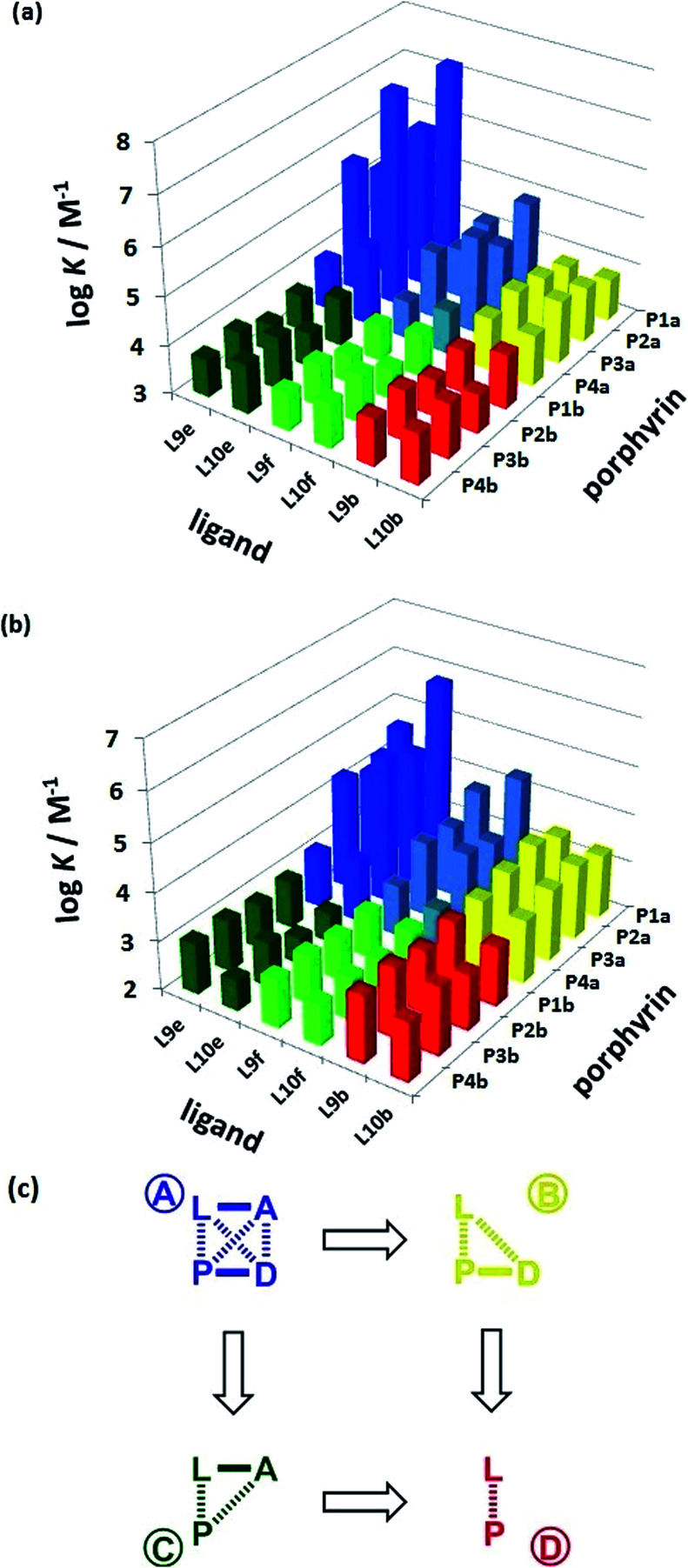
Association constants (log *K*/M^−1^) measured in (a) toluene and (b) TCE. The data are colour coded according to the role in the DMC. (c) Schematic representation of the chemical double mutant cycle used to extract information on the magnitude of the intramolecular H-bonding interaction between H-bond acceptor A and H-bond donor D in complexes formed between a zinc porphyrin (P) and a pyridine ligand (L).

The association constant data in Tables 1 and 2 are illustrated graphically in Fig. 6. The results are colour coded according to the role of the complex in the DMC. Complexes that can make an intramolecular H-bond (blue), are generally more stable than the complexes that cannot (yellow, green and red). The complexes containing ligands that can make a phenol-amide H-bond (Le, dark blue) are more stable than complexes containing ligands that can make a phenol-ester H-bond (Lf, pale blue), because amides are stronger H-bond acceptors. The free energy contributions of intramolecular H-bonds were determined using the data in Tables 1 and 2 and eqn (1). The results are listed in Tables 3 and 4 for toluene and TCE respectively. In toluene, 12 of 16 complexes make detectable intramolecular H-bonds. In TCE, 11 of 16 complexes make detectable H-bonds.

**Table 3:** Free energy contributions from amide-phenol and ester-phenol H-bonds (ΔΔ*G*^0^/kJ mol^−1^) determined using the chemical double mutant cycle in Fig. 2 at 298 K in toluene^a^

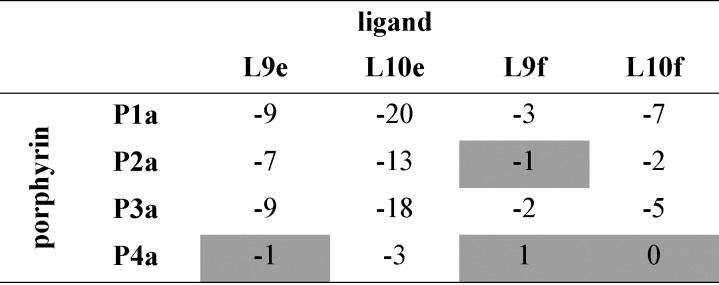					

a Average error over the data set is ±1 kJ mol^−1^. Complexes that do not make detectable H-bonds are shaded.

**Table 4:** Free energy contributions from amide-phenol and ester-phenol H-bonds (ΔΔ*G*^0^/kJ mol^−1^) determined using the chemical double mutant cycle in Fig. 2 at 298 K in TCE^a^

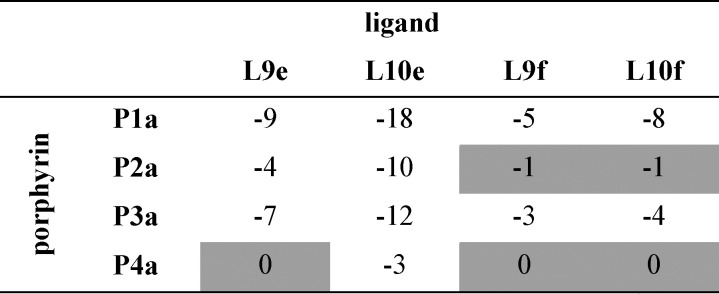					

a Average error over the data set is ±1 kJ mol^−1^. Complexes that do not make detectable H-bonds are shaded.

**Fig. 7:**
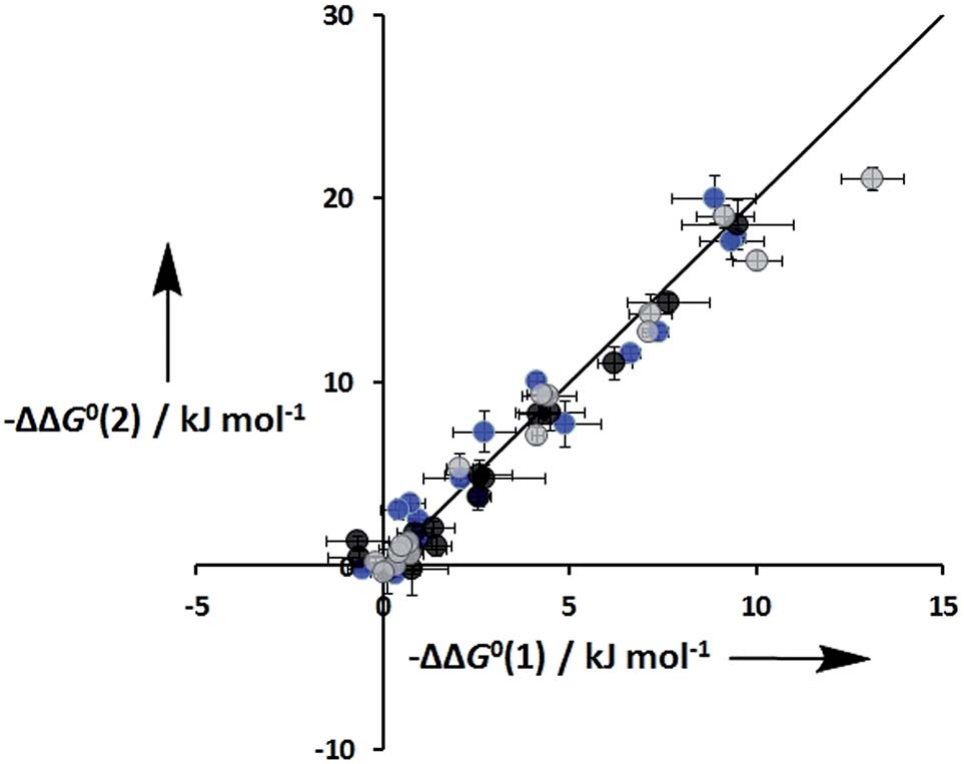
Total free energy contribution due to intra-molecular H-bonding for ligands with two identical side arms, ΔΔ*G*^0^(2), compared with data for the corresponding one-armed ligands, ΔΔ*G*^0^(1). Data for ligands with an ether linker are shown in blue (L9 and L10), ester linker in black (L2 and L3) and aromatic linker in grey (L7 and L8). The line corresponds to ΔΔ*G*^0^(2) = 2ΔΔ*G*^0^(1).

An inherent assumption of the DMC methodology is that the free energy contributions from individual interactions are additive. Fig. 7 compares the total free energy contribution due to H-bonding interactions in complexes of one-armed ligands, which can only make one H-bond, and complexes of the corresponding two armed ones, which can make two identical H-bonds. The free energy contribution due to two H-bonds, ΔΔ*G*^0^(2), is double the contribution of one H-bond, ΔΔ*G*^0^(1), in all of the systems studied here, confirming the validity of the additivity assumption.

**Fig. 8:**
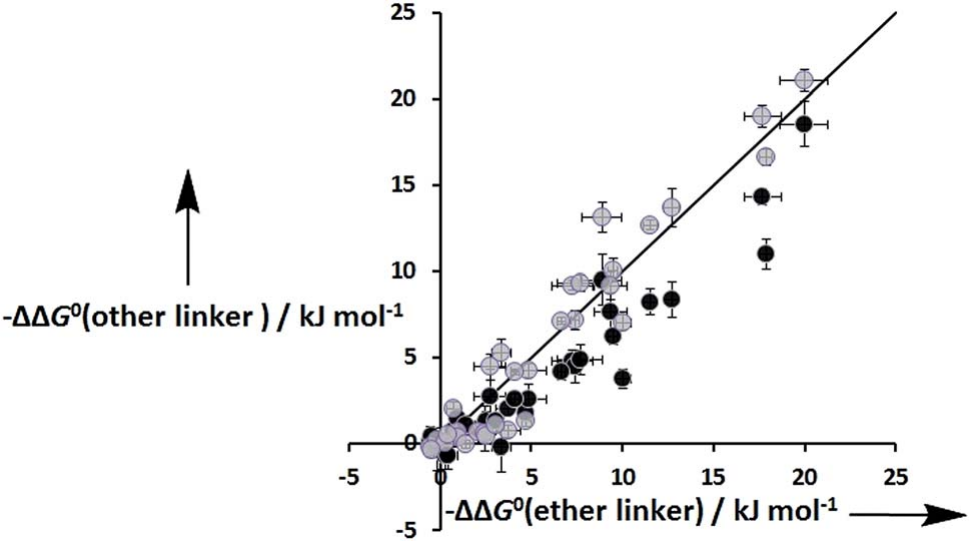
Total free energy contribution due to intra-molecular H-bonding for ligands with an ether linker, ΔΔ*G*^0^(ether linker), compared with data for the corresponding ligands with an ester linker (black) and aromatic linker (grey), ΔΔ*G*^0^(other linker). The line corresponds to ΔΔ*G*^0^(other linker) = ΔΔ*G*^0^(ether linker).

Fig. 8 shows that the free energy contributions due intramolecular H-bonds in complexes where the ligands have an ether linker are generally more favourable than for the corresponding interactions in complexes where the ligands have an ester linker. In contrast, the free energy contributions due to H-bonds in complexes where the ligands have an aromatic linker are practically identical to the corresponding complexes where the ligands with an ether linker. However, these free energy measurements are perturbed by differences in the intrinsic H-bond strength, which is perturbed by the linker, and in the degeneracies of the complexes. For the two armed ligands with the rigid aromatic linker (L8), formation of two H-bonds in the *cis* binding mode is geometrically impossible, so the degeneracy of the fully bound state is two. In contrast, the corresponding two-armed ester and ether linker ligands (L3 and L10) can form doubly H-bonded complexes in both the *cis* and *trans* binding modes, so the degeneracy of the fully bound state is six (see ESI for details†). Thus if we want to isolate the influence of linker flexibility on intramolecular H-bonding, we have to use the values of EM to remove the influence of these complicating factors.

### Effective molarities

**Fig. 9:**
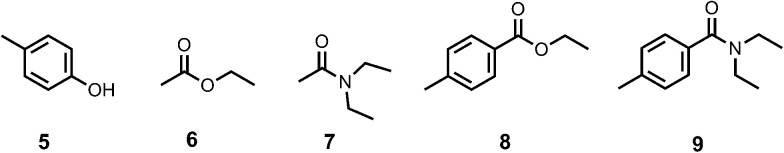
Compounds used to quantify intermolecular H-bonding interactions.

In order to determine the values of EM, the association constants for formation of the corresponding intermolecular interactions (*K*_ref_) were measured by ^1^H NMR titrations using the compounds shown in Fig. 9. In all cases, the data fit well to a 1 : 1 binding isotherm. The results are listed in Table 5 and compared with values estimated using literature H-bond parameters and eqn (3) (*K*_calc_).^14^

3 −*RT* ln *K*_calc_ = −(*α*_D_ − *α*_S_)(*β*_A_ − *β*_S_) + 6 kJ mol^−1^

**Table 5:** Association constants (M^−1^) for the formation of intermolecular H-bonded complexes measured by ^1^H NMR titrations in *d*_2_-TCE and *d*_8_-toluene at 298 K (*K*_ref_) and estimated using eqn (3) (*K*_calc_)

Solvent	Complex	*α*	*β*	*α*_s_	*β*_s_	*K*_ref_	*K*_calc_
TCE	5–6	3.8	5.4	2.0	1.3	2 ± 1	2
TCE	5–7	3.8	8.5	2.0	1.3	22 ± 3	16
TCE	5–8	3.8	5.1	2.0	1.3	2 ± 1	1
TCE	5–9	3.8	7.9	2.0	1.3	11 ± 2	11
Toluene	5–6	3.8	5.4	1.0	2.2	3 ± 1	3
Toluene	5–7	3.8	8.5	1.0	2.2	86 ± 20	110
Toluene	5–8	3.8	5.1	1.0	2.2	3 ± 1	2
Toluene	5–9	3.8	7.9	1.0	2.2	33 ± 1	54

There is a good agreement between the experimental and calculated values, and this confirms that the measurements of *K*_ref_ for the very weak phenol-ester H-bonds are reliable.

Complexes held together by multiple non-covalent interactions are actually a mixture of partially and fully bound states. For example, the complex shown in Fig. 1b is a mixture of a partially bound state, which only has the zinc–nitrogen coordination bond, and a fully bound state, which has both the coordination bond and the H-bond. The observed association constant, *K*_obs_, for this system would be the sum of the association constants of all of the bound states (eqn (4)).

4 *K*_obs_ = *K*_0_ + *K*_0_*K*_ref_EM = *K*_0_(1 + *K*_ref_EM)

For complexes studied here, there are multiple H-bonding sites, so statistical factors that account for the degeneracy of each partially bound state must also be included. For the one-armed ligand complexes formed with the Pa porphyrins, there are four possible H-bonding interactions, so the value of *K*_obs_ is given by eqn (5).

5 *K*_obs_ = *K*_0_(1 + 4*K*_ref_EM)

For the two-armed ligand complexes, we assume that EM for the formation of the second H-bond is the same as the value of EM for formation of the first H-bond. Thus the value of *K*_obs_ is given by eqn (6) for ligands with the aromatic linker and eqn (7) for ligands with the ester or ether linkers. The statistical factors are different for the aromatic linker, because these ligands can only form two H-bonds simultaneously in the *trans* binding mode, whereas the other ligands can form two H-bonds in both *cis* and *trans* binding modes.

6 *K*_obs_ = *K*_0_(1 + 8*K*_ref_EM + 4(*K*_ref_EM)^2^)

7 *K*_obs_ = *K*_0_(1 + 8*K*_ref_EM + 12(*K*_ref_EM)^2^)

The value of *K*_0_ varies with the structure of the ligand, but these differences cancel out in the DMC, so the values of EM can be calculated using eqn (8) for one-armed ligands, eqn (9) for two-armed ligands with the aromatic linker and eqn (10) for two-armed ligands with ester or ether linkers. The values of EM are shown in Tables 6 and 7 for measurements in toluene and in TCE respectively.

8 e^−ΔΔ*G*^0^/*RT*^ = 1 + 4*K*_ref_EM

9 e^−ΔΔ*G*^0^/*RT*^ = 1 + 8*K*_ref_EM + 4(*K*_ref_EM)^2^

10 e^−ΔΔ*G*^0^/*RT*^ = 1 + 8*K*_ref_EM + 12(*K*_ref_EM)^2^

**Table 6:** Effective molarities (EM/mM) for intramolecular amide-phenol and ester-phenol H-bonds measured at 298 K in toluene^a^

		Ligand			
		L9e	L10e	L9f	L10f
Porphyrin	P1a	100	180	170	310
	P2a	55	40	b	57
	P3a	130	120	110	150
	P4a	b	3	b	b

a Average error over the data set is ±50%.

b No interaction detected.

**Table 7:** Effective molarities (EM/mM) for intramolecular amide-phenol and ester-phenol H-bonds measured at 298 K in TCE^a^

		Ligand			
		L9e	L10e	L9f	L10f
Porphyrin	P1a	480	450	770	520
	P2a	48	85	b	b
	P3a	150	120	220	150
	P4a	b	10	b	b

a Average error over the data set is ±50%.

b No interaction detected.

**Fig. 10:**
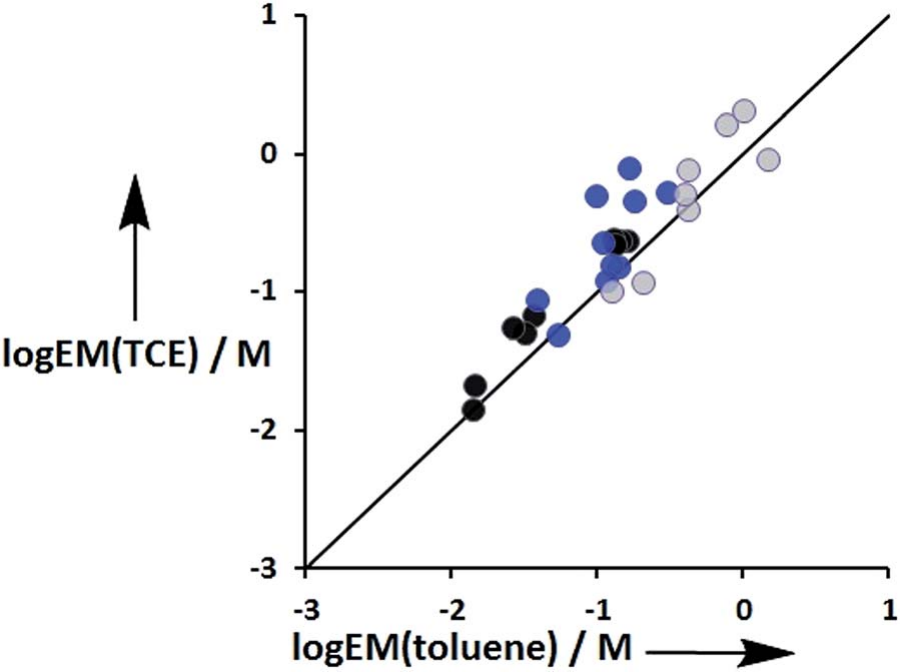
Comparison of effective molarities (EM) for formation of intramolecular H-bonds in toluene with the corresponding values measured in TCE. Data for ligands with an ether linker are shown in blue (L9 and L10), ester linker in black (L2 and L3) and aromatic linker in grey (L7 and L8).^9^ The line corresponds to EM(TCE) = EM(toluene).

The values of EM vary from 3 mM to 770 mM. Fig. 10 compares values of EM measured in toluene with the corresponding values measured in TCE. There is good agreement between data obtained in the two different solvents, which indicates that EM is independent of solvent. This might be expected, because the major influence of solvent in these systems is to change the intrinsic strength of the individual interactions, which is factored out by the EM analysis.

**Fig. 11:**
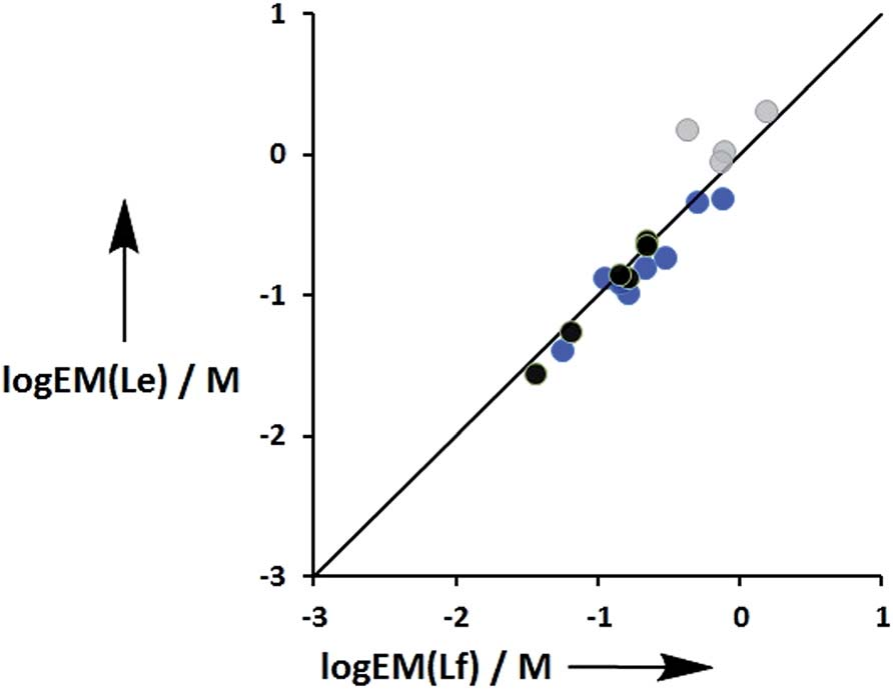
Comparison of effective molarities (EM) for formation of intramolecular phenol-amide H-bonds, log EM(Le), with the corresponding values measured for phenol-ester H-bonds, log EM(Lf). Data for ligands with an ether linker are shown in blue (L9 and L10), ester linker in black (L2 and L3) and aromatic linker in grey (L7 and L8).^9^ The line corresponds to log EM(Le) = log EM(Lf).

Fig. 11 compares the values of EM measured for phenol-amide H-bonds using the Le ligands with the values of EM measured for phenol-ester H-bonds using the Lf ligands. The values of EM are practically identical for the two different H-bond acceptors. There is a significant difference between the strengths of amide and ester H-bonds (one order of magnitude in the association constant), so these results indicate that EM depends only on the supramolecular architecture and is independent of the intrinsic strength of the individual interactions.

**Fig. 12:**
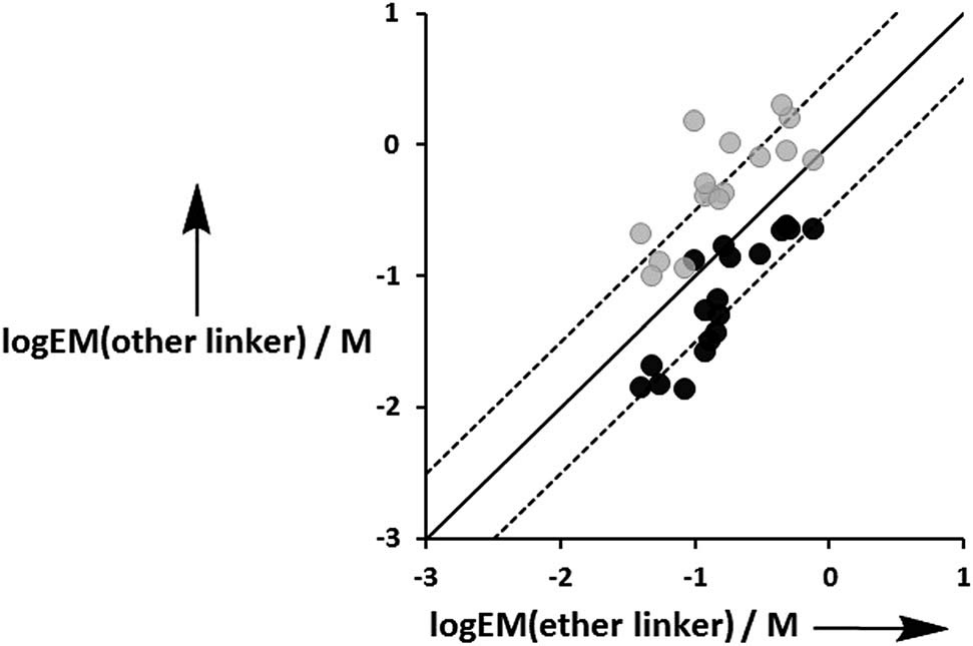
Comparison of effective molarities (EM) measured for formation of intramolecular H-bonds for ligands L9 and L10, log EM(ether linker), with the values measured for the corresponding ligands with an ester linker (L2 and L3 in black) and aromatic linker (L7 and L8 in grey), log EM(other linker). The solid line corresponds to log EM(other linker) = log EM(ether linker), and the dashed lines correspond to log EM(other linker) = log EM(ether linker) ± 0.5.

Fig. 12 compares the values of EM measured for the three different types of linker. Although there is considerable scatter in the data, the values of EM for complexes with the aromatic linker are generally higher than the corresponding values measured for the more flexible ether linker by a factor of about 3. However, the values of EM for complexes with the ester linker are generally lower than the corresponding values for the more flexible ether linker, again by a factor of about 3. These results indicate that the relationship between conformational flexibility and EM is not straightforward: there is a trade off between the ability of flexible ligands to optimize geometric complementarity, which improves binding affinity, and restriction of conformational degrees of freedom, which reduces binding affinity. Thus the most rigid ligands, L7f and L8f, which have aromatic linkers, do not make detectable H-bonds with P3a, whereas the corresponding flexible ligands, L9f and L10f, which have ether linkers, make H-bonds worth 2 to 5 kJ mol^−1^. When geometric complementarity is more optimal, the most rigid ligands, which have aromatic linkers, make the strongest H-bonds with the highest values of EM: for example, the EM values for the complexes formed between P3a and the most rigid ligands, L7e and L8e, which have aromatic linkers, are 380–500 mM compared with 120–160 mM for the most flexible ligands, L9e and L10e, which have ether linkers.

## Conclusions

The thermodynamic properties of a family of 48 new zinc porphyrin–pyridine complexes have been compared with closely related complexes described previously. Chemical double mutant cycles were used to dissect the contributions of intramolecular H-bonds in 96 different complexes. Comparison of the free energy contributions from the DMCs with association constants for the corresponding intermolecular interactions was used to determine the values of EM, which quantify chelate cooperativity in these systems. The values of EM vary with the degree of conformational flexibility of the ligands.^8*c*^ The values of EM for the most flexible ligands, which have ether linkers, lie in between the values of EM for two sets of more rigid ligands with aromatic and ester linkers. The most rigid ligands make high affinity complexes with the porphyrin receptors only when there is good geometric complementarity.^9^ More flexible ligands can adapt their conformation to optimize interactions where geometric complementarity is poor, and this results in more stable complexes than found for the rigid ligands. The advantage of induced fit geometric complementarity and the disadvantage of restriction of conformational degrees of freedom in flexible ligands compete to determine the overall effect of conformational flexibility on binding affinity.^7*a*^ Flexible systems are usually easier to synthesize than highly preorganized rigid molecules, and the results presented here suggest that the additional synthetic effort required to prepare highly preorganized systems may not be a rewarding strategy in supramolecular design. Flexible molecules, which adapt to the optimum conformation upon complexation, provide a good alternative for building supramolecular systems with high binding affinity.

## Experimental

### Synthesis

#### Ligand L9e

Sodium hydride (60% dispersion in mineral oil, 0.42 g, 10.5 mmol) was added to *N*,*N*-diethyl-2-hydroxy-acetamide (1.24 mL, 9.53 mmol) in anhydrous tetrahydrofuran (60 mL) at 0 °C. The reaction suspension was stirred for 30 minutes at 0 °C and then 3-(bromomethyl)pyridine hydrobromide (2.41 g, 9.53 mmol) was added in portions. The reaction mixture was allowed to warm to room temperature over 1.5 hours and then quenched with water (100 mL). The reaction mixture was then extracted with ethyl acetate (2 × 200 mL), and the combined organic layers were washed with water (100 mL), brine (30 mL) and dried with magnesium sulfate. The solvent was removed on a rotary evaporator, and the residue was purified on silica eluting with dichloromethane–methanol (100 : 0 to 95 : 5). The product was isolated as a colorless oil (0.19 g, 9%); ^1^H NMR (400 MHz, CDCl_3_): *δ*_H_ = 8.54 (d, 1H, *J* = 2), 8.49 (dd, 1H, *J* = 5, *J* = 2), 7.70 (dt, 1H, *J* = 8, *J* = 2), 7.24 (dd, 1H, *J* = 8, *J* = 5), 4.59 (s, 2H), 4.14 (s, 2H), 3.33 (q, 2H, *J* = 7), 3.21 (q, 2H, *J* = 7), 1.09 (t, 3H, *J* = 7), 1.08 (t, 3H, *J* = 7); ^13^C NMR (100 MHz, CDCl_3_): *δ*_C_ = 167.8, 149.2, 149.2, 135.8, 133.1, 123.4, 70.6, 69.1, 41.1, 40.1, 14.2, 12.9; HRMS (ES+): calcd for C_12_H_19_N_2_O_2_: 223.1447; found 223.1441; FT-IR (thin film): *ν*_max_/cm^−1^ 1639, 1429, 1381, 1269, 1220, 1114, 1030, 798, 714.

#### Ligand L10e

Sodium hydride (60% dispersion in mineral oil, 0.19 g, 4.68 mmol) was added to *N*,*N*-diethyl-2-hydroxy-acetamide (0.51 mL, 3.90 mmol) in anhydrous tetrahydrofuran (40 mL) at 0 °C. The reaction suspension was stirred for 20 minutes at 0 °C and then 3 (0.27 g, 0.78 mmol) was added slowly. The reaction mixture was allowed to warm to room temperature, stirred overnight. The solvent was removed on a rotary evaporator and the residue was dissolved in ethyl acetate (150 mL), washed with water (15 mL), brine (30 mL), dried with sodium sulfate. The solvent was removed on a rotary evaporator, and the residue was purified on silica eluting with ethyl acetate–ethanol (100 : 0 to 90 : 10). The product was isolated as a yellow oil (0.1 g, 35%); ^1^H NMR (400 MHz, CDCl_3_): *δ*_H_ = 8.50 (d, 2H, *J* = 2), 7.76–7.44 (m, 1H), 4.62 (s, 4H), 4.16 (s, 4H), 3.35 (q, 4H, *J* = 7), 3.23 (q, 4H, *J* = 7), 1.11 (t, 6H, *J* = 7), 1.10 (t, 6H, *J* = 7); ^13^C NMR (100 MHz, CDCl_3_): *δ*_C_ = 167.8, 147.8, 136.1, 133.5, 70.4, 69.2, 41.1, 40.2, 14.3, 12.9; HRMS (ES+): calcd for C_19_H_32_N_3_O_4_: 366.2393; found 366.2408; FT-IR (thin film): *ν*_max_/cm^−1^ 2972, 2934, 2874, 1636, 1460, 1433, 1380, 1350, 1265, 1219, 1010.

#### Ligand L9f

Sodium hydride (60% dispersion in mineral oil, 1.04 g, 26.1 mmol) was added to ethyl glycolate (1.48 g, 14.2 mmol) in anhydrous tetrahydrofuran (90 mL) at 0 °C. The reaction suspension was stirred for 30 minutes at 0 °C and then 3-(bromomethyl)pyridine hydrobromide (3.00 g, 11.9 mmol) was added in portions. The reaction mixture was allowed to warm to room temperature, stirred for 16 hours and then quenched with water (50 mL). The reaction mixture was then extracted with ethyl acetate (2 × 150 mL) and the combined organic layers were washed with water (30 mL), brine (30 mL), dried with magnesium sulfate. The solvent was removed on a rotary evaporator and the residue was purified on silica eluting with hexane–ethyl acetate (100 : 0 to 60 : 40). The product was isolated as a colorless oil (0.97 g, 42%); ^1^H NMR (400 MHz, CDCl_3_): *δ*_H_ = 8.58 (d, 1H, *J* = 2), 8.54 (dd, 1H, *J* = 5, *J* = 2), 7.74 (dt, 1H, *J* = 8, *J* = 2), 7.29 (dd, 1H, *J* = 8, *J* = 5), 4.64 (s, 2H), 4.22 (q, 2H, *J* = 7), 4.12 (s, 2H), 1.28 (t, 3H, *J* = 7); ^13^C NMR (100 MHz, CDCl_3_): *δ*_C_ = 169.9, 149.3, 149.2, 135.6, 132.6, 123.3, 70.7, 67.4, 60.8, 14.0; HRMS (ES+): calcd for C_10_H_14_NO_3_: 196.0974; found 196.0966; FT-IR (thin film): *ν*_max_/cm^−1^ 1748, 1579, 1427, 1388, 1271, 1207, 1126, 1028, 794, 713.

#### Ligand L10f

Sodium hydride (60% dispersion in mineral oil, 0.58 g, 14.4 mmol) was added to ethyl glycolate (1.25 g, 12.0 mmol) in anhydrous tetrahydrofuran (50 mL) at 0 °C. The reaction suspension was stirred for 20 minutes at 0 °C and then 3 (0.72 g, 2.08 mmol) was added slowly. The reaction mixture was allowed to warm to room temperature, and stirred overnight. The solvent was removed under on a rotary evaporator and the residue was dissolved in ethyl acetate (150 mL), washed with water (15 mL), brine (30 mL), dried with sodium sulfate. The solvent was removed on a rotary evaporator and the residue was purified on silica eluting with hexane–ethyl acetate (50 : 50 to 5 : 95). The product was isolated as a colourless oil (0.35 g, 54%); ^1^H NMR (400 MHz, CDCl_3_): *δ*_H_ = 8.51 (d, 2H, *J* = 2), 7.77–7.75 (m, 1H), 4.63 (s, 4H), 4.20 (q, 4H, *J* = 7), 4.10 (s, 4H), 1.26 (t, 6H, *J* = 7); ^13^C NMR (100 MHz, CDCl_3_): *δ*_C_ = 170.0, 148.8, 135.3, 132.7, 70.7, 67.7, 61.0, 14.2; HRMS (ES+): calcd for C_15_H_22_NO_6_: 312.1447; found 312.1443; FT-IR (thin film): *ν*_max_/cm^−1^ 2983, 1747, 1582, 1433, 1384, 1274, 1202, 1119, 1025, 712.

#### Ligand L9b

Sodium hydride (60% dispersion in mineral oil, 1.00 g, 26.9 mmol) was added to ethanol (1.20 mL, 20.2 mmol) in anhydrous tetrahydrofuran (50 mL) at 0 °C under the protection of nitrogen. This reaction suspension was stirred for 20 minutes at 0 °C and then 3-(bromomethyl)pyridine hydrobromide (1.70 g, 6.72 mmol) was added slowly. The reaction mixture was allowed to warm to room temperature, stirred overnight. The solvent was removed on a rotary evaporator and the residue was dissolved with ethyl acetate (150 mL), washed with water (15 mL), brine (30 mL), dried with sodium sulfate. The solvent was removed on a rotary evaporator and the residue was purified on silica eluting with hexane–ethyl acetate (55 : 45 to 45 : 55). The product was isolated as a yellow oil (0.43 g, 47%); ^1^H NMR (400 MHz, CDCl_3_): *δ*_H_ = 8.54 (d, 1H, *J* = 2), 8.50 (dd, 1H, *J* = 5, *J* = 2), 7.65 (dt, 1H, *J* = 8, *J* = 2), 7.24 (dd, 1H, *J* = 8, *J* = 5), 4.48 (s, 2H), 3.53 (q, 2H, *J* = 7), 1.22 (t, 3H, *J* = 7); ^13^C NMR (100 MHz, CDCl_3_): *δ*_C_ = 149.1, 149.0, 135.3, 134.0, 123.3, 70.1, 66.1, 15.1; HRMS (ES+): calcd for C_8_H_12_NO: 138.0919; found 138.0918; FT-IR (thin film): *ν*_max_/cm^−1^ 2976, 2867, 1578, 1479, 1427, 1374, 1094, 1027, 789, 712.

#### Ligand L10b

Sodium hydride (60% dispersion in mineral oil, 1.37 g, 34.3 mmol) was added to ethanol (1.70 mL, 29.4 mmol) in anhydrous tetrahydrofuran (50 mL) at 0 °C under the protection of nitrogen. The reaction suspension was stirred at 0 °C for 20 minutes and then 3 (1.68 g, 4.90 mmol) was added slowly. The reaction mixture was allowed to warm to room temperature, stirred overnight. The solvent was removed on a rotary evaporator and the residue was dissolved in ethyl acetate (150 mL), washed with water (15 mL), brine (30 mL), dried over sodium sulfate. The solvent was removed on a rotary evaporator and the residue was purified on silica eluting with hexane–ethyl acetate (60 : 40 to 15 : 85). The product was isolated as a colorless oil (0.28 g, 29%); ^1^H NMR (400 MHz, CDCl_3_): *δ*_H_ = 8.48 (d, 2H, *J* = 2), 7.66 (s, 1H), 4.50 (s, 4H), 3.54 (q, 4H, *J* = 7), 1.23 (t, 6H, *J* = 7); ^13^C NMR (100 MHz, CDCl_3_): *δ*_C_ = 148.3, 134.7, 133.8, 70.0, 66.1, 15.1; HRMS (ES+): calcd for C_11_H_18_NO_2_: 196.1338; found 196.1329; FT-IR (thin film): *ν*_max_/cm^−1^ 2975, 2864, 1582, 1434, 1373, 1351, 1161, 1095, 1030, 712.

### Automated UV/Vis absorption titrations

UV/Vis titrations were carried out using a BMG FLUOstar Omega plate reader equipped with a UV/Vis detector and equilibrated at 298 K. A 5 mL solution of porphyrin was prepared at known concentration (1–5 μM) in spectroscopic grade solvent. A 10 mL solution of ligand was prepared at known concentration (8–40 000 μM) using spectroscopic grade solvent. 150 μL of the porphyrin solution was added to a well of a Hellma quartz microplate, and the absorbance at five wavelengths was recorded. Aliquots of the ligand solution (3, 6 or 10 μL) were successively added to the well, and the absorbance was recorded after each addition. Changes in absorbance were fit to a 1 : 1 binding isotherm in Microsoft Excel to obtain the association constant. Each titration was repeated at least three times, and the experimental error is quoted as twice the standard deviation at a precision of one significant figure.

### Manual fluorescence titrations

Fluorescence titrations were carried out using a Hitachi F-4500 Fluorescence Spectrophotometer at 298 K. A 10 mL solution of porphyrin at known concentration (0.04–0.05 μM) was prepared in spectroscopic grade solvent. Then, 2 mL of this host solution was loaded into a 1 cm path length fluorescence cuvette, and the fluorescence emission spectra was recorded from 500 to 750 nm exciting at 427 nm. A 2 mL solution of ligand (0.1–1 μM) was prepared using the host stock solution, so that the concentration of host remained constant throughout the titration. Aliquots of ligand solution were added successively to the cuvette, and the emission spectrum was recorded after each addition. Changes in fluorescence emission were fit to a 1 : 1 binding isotherm in Microsoft Excel to obtain the association constant. Each titration was repeated at least three times, and the experimental error is quoted as twice the standard deviation at a precision of one significant figure.

## Supplementary Material

SC-006-C4SC03398A-s001
