# Supplementary material for: The flexibility–complementarity dichotomy in receptor–ligand interactions
Source: Chem Sci. 2014 Dec 15;6(2):1444–53. doi: 10.1039/c4sc03398a (PMC5811160; doi:10.1039/c4sc03398a)
Supplement: SC-006-C4SC03398A-s001 [file SC-006-C4SC03398A-s001.pdf]

Electronic Supplementary Material (ESI) for Chemical Science.
This journal is © The Royal Society of Chemistry 2014

The Flexibility-Complementarity Dichotomy in Receptor-Ligand Interactions

Hongmei Sun,[a] Christopher A. Hunter,* [a,b] and Eva Marina Llamas [a]

[a] *Department of Chemistry, University of Sheffield, Sheffield S3 7HF (UK)*

[b] *Department of Chemistry, University of Cambridge, Lensfield Road, Cambridge CB2 1EW (UK)*

Supplementary Information

Partially bond states analysis

The observed association constant for a complex that makes multiple intermolecular interactions is given by summing over all partially and fully bound states. The observed association constant K_{obs} is given by equation (S1)

$$K_{\text{obs}} = fK_0 \quad \text{Eq. S1}$$

where K_0 is the association constant for formation of only the zinc-nitrogen coordination bond, and f depends on the number and type of H-bonds that are made as well as the overall symmetry of the complex as given by equation (S2)

$$f = 1 + \sum_i \sigma_i K_i EM_i + \sum_{i,j}^{N} \sigma_{ij} K_i EM_i K_j EM_j + \cdots + \sigma_{ij\cdots N} \prod_i^N K_i EM_i \quad \text{Eq. S2}$$

Where K_i is the association constant for formation of the corresponding intermolecular H-bond (or K_{ref}), EM$_i$ is the effective molarity or formation of the intramolecular H-bond, and σ_i are statistical factors that account for the degeneracies of the different bound states. The zinc-nitrogen coordination bonds are not identical in all of the complexes, but differences in K_0 cancel out in the DMC, so that the value of ΔΔG° can be related to the values of K_i and EM$_i$ by equation (S3). There are two different classes of complexes studied, and the details of the equations used for f in each of these systems are provided below.

$$e^{-\Delta\Delta G^\circ/RT} = \frac{f_A f_D}{f_B f_C} \quad \text{Eq. S3}$$

Complexes that make one H-bond

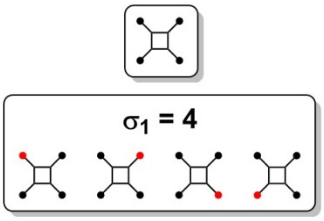

Figure S1. Degenerate states for the complex that can make one intramolecular H-bond. Unbound phenol OH groups on the porphyrin are represented by black dots, phenol groups H-bonded to an ester group, amide group or a phosphine oxide group are represented by red dots. The panels are ordered zero and one H-bond from top to bottom and grouped by symmetry.

For complexes that can make only one intramolecular H-bond, the observed association constant for complex A (Figure 2) is given by equation S4, while the observed association constant for complex B, C, and D (Figure 2), where no H-bonds are made, is given by equation S5. The value of K_1EM_1, thus the value of EM_1, can be determined from the relevant value of $\Delta\Delta G^0$ using equation S6 and S7.

$$f_A = 1 + \sigma_1 K_1 EM_1 \quad \text{Eq. S4}$$

$$f_B = f_C = f_D = 1 \quad \text{Eq. S5}$$

$$e^{-\frac{\Delta\Delta G^0}{RT}} = \frac{f_A f_D}{f_B f_C} = 1 + \sigma_1 K_1 EM_1 \quad \text{Eq. S6}$$

$$K_1 EM_1 = \frac{e^{-\frac{\Delta\Delta G^0}{RT}} - 1}{\sigma_1} \quad \text{Eq. S7}$$

where K_1 is the intermolecular association constant for formation of the H-bonding interaction (K_{ref} in Table 7), EM_1 is the effective molarity for the intramolecular interaction, and σ_1 is the statistical factor that accounts for the degeneracy of the fully bound state. In the complexes discussed here, the hydroxyporphyrins all have four identical H-bond donor sites, so $\sigma_1 = 4$ (Figure S1).

Complexes that make two identical H-bonds

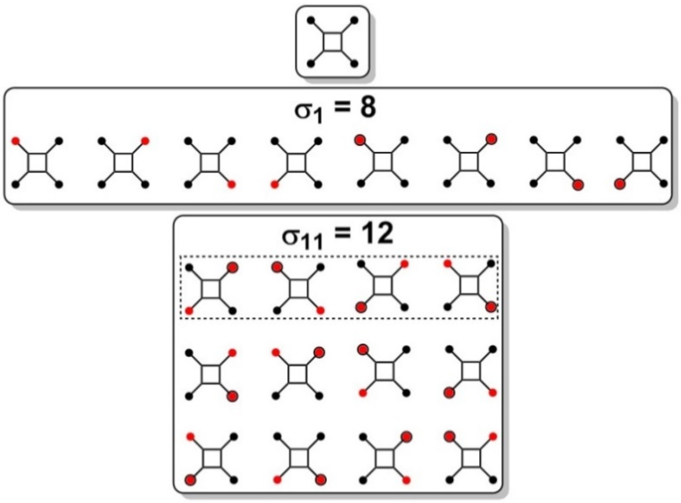

Figure S2. Degenerate states for the complex that can make two identical intramolecular H-bonds. Unbound phenol OH groups on the porphyrin are represented by black dots, phenol groups H-bonded to ester groups, amide groups or phosphine oxide groups are represented by red dots. The panels are ordered zero, one and two H-bonds from top to bottom and grouped by symmetry. The *trans* arrangements are highlighted by the dotted box.

For complexes that can make two identical intramolecular H-bonds, the observed association constant for complex A (Figure 2) is given by equation S8 , while the observed association constant for complex B (Figure 2), where no H-bonds are made, is given by equation S9. The value of K_1EM_1 can be obtained from the relevant value of $\Delta\Delta G^0$ using equation S10 and S11. We assume that the values of K_1EM_1 for the first and the second H-bonds are constant. The fact that $\Delta\Delta G^0$ for the two armed ligands is always twice that for the one armed ligands supports this assumption.

$$f_A = 1 + \sigma_1 K_1 EM_1 + \sigma_{11}(K_1EM_1)^{\ 2} \qquad \text{Eq. S8}$$

$$f_B = f_C = f_D = 1 \qquad \text{Eq. S9}$$

$$e^{-\frac{\Delta\Delta G^0}{RT}} = \frac{f_A f_D}{f_B f_C} = 1 + \sigma_1 K_1 EM_1 + \sigma_{11}(K_1EM_1)^2 \qquad \text{Eq. S10}$$

$$K_1EM_1 == \frac{-\sigma_1 + \sqrt{{\sigma_1}^2 - 4\sigma_{11}\left(1 - e^{-\frac{\Delta\Delta G^0}{RT}}\right)}}{2\sigma_{11}} \quad \text{Eq. S11}$$

For two armed ligands with aromatic linkers (**L8**), they can only form two H-bonds simultaneously in the *trans* binding modes with **Pa** porphyrins (highlighted by the dotted box in Figure S2**)**, so $\sigma_1 = 8$ and $\sigma_{11} = 4$.

For two armed ligands with ester linkers (**L3**) or ether linkers (**L10**), they can form two H-bonds with **Pa** porphyrins in both *trans* and *cis* binding modes, so $\sigma_1 = 8$ and $\sigma_{11} = 12$.
